# Comparison of the indocyanine green dye method versus the combined method of indigo carmine blue dye with indocyanine green fluorescence imaging for sentinel lymph node biopsy in breast conservative therapy for stage ≤IIA breast cancer

**DOI:** 10.1186/s12905-018-0646-5

**Published:** 2018-09-18

**Authors:** Nobuyuki Takemoto, Ai Koyanagi, Masanori Yasuda, Hiroshi Yamamoto

**Affiliations:** 1 Department of Breast & Endocrine Surgery, Japan Medical Alliance East Saitama General Hospital, 5-517, Yoshino, Satte, Saitama, 0153-340 Japan; 2 Research and Development Unit, Parc Sanitari Sant Joan de Déu, Fundació Sant Joan de Déu, CIBERSAM, Sant Boi de Llobregat, Barcelona, Spain; 3 Department of Pathology, Saitama Medical University International Medical Center, Hidaka, Saitama, Japan; 4 Geriatric Health Service Facility (COSMOS), Japan Medical Alliance Yokohama Stroke and Brain Center, Yokohama city, Japan

**Keywords:** Sentinel lymph node biopsy, Identification rate, Indocyanine green (ICG), Indigo carmine, Fluorescence, Breast cancer

## Abstract

**Background:**

Fluorescence imaging (FI) is one of the methods to identify sentinel lymph nodes (SLNs). However, the procedure is technically complicated and requires procedural skills, as SLN biopsy must be conducted in dim light conditions. As an improved version of this method, we introduced a combined method (Combined mixed dye and fluorescence; CMF) consisting of indigo carmine blue dye and FI. The direct visualization of SLNs under shadowless surgical light conditions is facilitated by the addition of the blue dye. We compared the SLN detection rates of CMF with that of the indocyanine green (ICG) dye method (ICG-D).

**Methods:**

A total of 202 patients with stage ≤IIA breast cancer who underwent breast conservative therapy with separate incision from January 2004 to February 2017 were reviewed. Details of the two methods are as follows: (1) ICG-D: 10 mg of ICG was used and the green-stained SLNs were resected via a 3-4 cm axillary incision; (2) CMF: A combination of 5 mg of ICG and 4-8 mg of indigo carmine was used. After a 1.5–2 cm incision was made near the point of disappearance of the fluorescence using Photodynamic Eye (PDE), the blue-stained SLNs were resected under shadowless surgical light conditions.

**Results:**

There were 92 ICG-D and 110 CMF cases. CMF resulted in a significantly higher SLN detection rate than ICG-D (96.4% vs. 83.7%; *p* = 0.003). This difference was particularly notable in those aged ≥60 years (98.3% vs. 74.3%) and individuals with body mass index (BMI) ≥25 kg/m^2^ (90.3% vs. 58.3%).

**Conclusion:**

CMF is an effective method to identify SLNs which is safe and efficient. CMF achieves a high SLN identification rate and most of this procedure is feasible under shadowless surgical light conditions. CMF can reliably perform SLN biopsy even in those aged ≥60 years and individuals with BMI ≥ 25 kg/m^2^.

## Background

The lymphatic channel network in the breast is developed mainly around the areola and the main export route is towards the axilla. The sentinel lymph nodes (SLNs), which are the first accumulation point of this lymphatic flow, are the forefront of the immune surveillance mechanism in the breast. In terms of surgical treatments for breast cancer, in the absence of SLN metastasis, it has been reported that the omission of axillary dissection does not affect prognosis [1–3].

Currently, there exists several methods for the identification and biopsy of SLNs (e.g., dye method, radioisotope (RI) method, and a combination of these two) [4]. The benefits of the dye method are its simplicity and cost effectiveness [5, 6]. However, this method requires a learning curve to acquire procedural skills [5–9], and the location of the incision mainly depends on experience as the blue dye cannot be seen through the skin [10]. Next, in terms of the RI method, although this allows for the identification of the precise location of SLNs, patient radiation exposure cannot be avoided [10, 11], while it cannot be performed in institutes without nuclear medicine facilities [10, 12, 13]. As for the identification rate of SLNs, it has been reported that the combined method of dye and RI performs best [14, 15]. However, drawbacks of the RI method remain unchanged.

The fluorescence method using indocyanine green (ICG: Daiichi-Sankyo, Tokyo Japan) has been proposed as an alternative to both dye and RI methods [11, 16]. ICG is a widely used reagent for liver function tests. The excitation wavelength of ICG is between 750 nm to 810 nm, and a fluorescence peak wavelength is generated at 845 nm when it is combined with plasma protein [17]. Although the fluorescence of ICG cannot be directly visualized with the naked eye, it can be confirmed on the monitor in real-time through an equipment required for near-infrared fluorescence imaging [e.g., Photodynamic Eye (PDE) (Hamamatsu Photonics Co., Hamamatsu, Japan)] [12, 18], which is much less costly than the establishment of RI facilities. Thus, this method can be performed under the current state of many countries worldwide where RI facilities are limited. This method also allows for real-time intra-operative identification of SLNs and lymphatic flows without specialized training [4, 8, 12, 19]. Specifically, the fluorescence of the lymphatic channels is first confirmed and traced until the first-drained lymph node in the axilla is identified through the monitor. Thus, it is possible to reliably identify SLNs, and obtain high detection and low false-negative rates [19, 20] which is comparable to those of the combined method of radioisotope and blue dye [15, 21]. However, this method is not exempt from drawbacks either. For example, the operation must be performed in dim light conditions to confirm the fluorescence via a monitor [16, 19, 22, 23], while operation time is limited due to the fast spread of the ICG [4, 9, 16, 19].

In order to overcome the weaknesses of the above-mentioned methods, in 2011, we introduced the combined mixed dye and fluorescence (CMF) method which is similar to the fluorescence method using ICG but mixes blue dye (indigo carmine: Daiichi-Sankyo, Tokyo Japan) into ICG. This method maintains the advantages of the fluorescence method, but further simplifies the procedure. Specifically, its benefits are associated with the direct visualization of SLNs under a shadowless surgical light, which is made possible for the addition of the blue dye. Thus, the main objective of this study was to compare the SLN detection rates between the CMF method and the dye method using ICG (ICG-D) in a single facility in Japan.

## Methods

### Patients

Patients with early breast cancer who underwent Breast Conservative Therapy (BCT) between December 2003 and February 2017 were reviewed retrospectively. Indication for BCT was based on the guidelines of BCT issued by the Japanese Breast Cancer Society [24]. Patients with primary tumor size > 3 cm, multicentric breast cancer, and tumors fixed to the skin or muscle were not subject to BCT. Our analysis was restricted to those who had an indication for SLN biopsy (i.e., ≤T2 breast cancer with clinically negative axillary lymph nodes) based on preoperative examinations (mammography, ultrasound, enhanced multi-detector computed tomography, and enhanced magnetic resonance imaging, etc). Furthermore, in this study, only cases that underwent BCT with separate incisions (incision on the breast for tumor resection and a small axillar incision for SLN biopsy) were reviewed. Patients who had neoadjuvant chemotherapy, history of allergy to iodine, or history of prior breast and axillary surgery were excluded.

The main method for identification of SLNs for SLN biopsy was the ICG-D method until November 2011, while the CMF method was introduced in December 2011 after approval by the ethics committee of East Saitama General Hospital. There were no overlaps in the period in which ICG-D and CMF were performed. All resected SLNs were pathologically examined by hematoxylin and eosin stain. Pathological examinations were conducted by pathologists with more than 20 years’ experience. Until April 2008, all pathological diagnoses of SLN biopsy specimens were performed on permanent sections. In positive SLN cases, an additional axillary lymph node dissection was performed at a later date. After April 2008, intra-operative examination of SLN biopsy specimens became available, and was performed with one or more frozen sections. Positive SLNs in the frozen section resulted in additional axillary lymph node dissection on-spot. Additional axillary dissection of the lymph nodes was performed on a later date if the frozen section was negative but the permanent section was positive. This was not conducted if the diagnosis according to the permanent section was micrometastasis. For both ICG-D and CMF, axillary lymph node dissection was added when the SLN could not be identified. Post-operatively, a histopathologic examination was also performed on the resected specimens to confirm the diagnosis. Further examinations such as estrogen and progesterone receptor and herceptine score (HER2) were also conducted. If the herceptine score was + 2, the Fish method was added, and a final HER2-positive diagnosis was made when confirmed to be FISH positive. With the exception of 4 ICG-D cases, all operations were performed by the same operator (NT) who already had more than 14 years of experience in breast surgery when the first ICG-D in this study was performed (2004). In the 4 exceptional cases, the main surgeon had more than 10 years of experience while NT assisted the operation.

All patients in the study underwent tangential field irradiation of the whole breast (50Gy), with a dose per fraction of 2Gy in 5 weeks post-operatively. If margins were close to the tumor edge (≤5 mm), a boost of 10Gy was added. Indication for post-operative chemotherapy and hormone therapy was based on the St. Gallen International Expert Consensus at the time of the operation. Detailed information on all surgical procedures were provided to all patients and family members preoperatively and written informed consent was obtained.

### ICG-D method

The patient was placed in a supine position on the operating table and a small pillow was placed on the back of the operating side of the patient. The arm on the surgical side was extended perpendicular to the body axis. After the induction of general anesthesia and sterilization of the operation site, ICG 10 mg, diluted with 2 ml of distilled water, was injected into the sub-areolar region after induction of general anesthesia. The sub-areolar region was compressed to assess lymphatic drainage and a 3-4 cm incision was made at the axillar region and the green-stained SLNs were resected under shadowless surgical light conditions. After SLN biopsy, partial resection of the mammary gland was performed through a separate incision in succession.

### CMF method

**Fig. 1:**
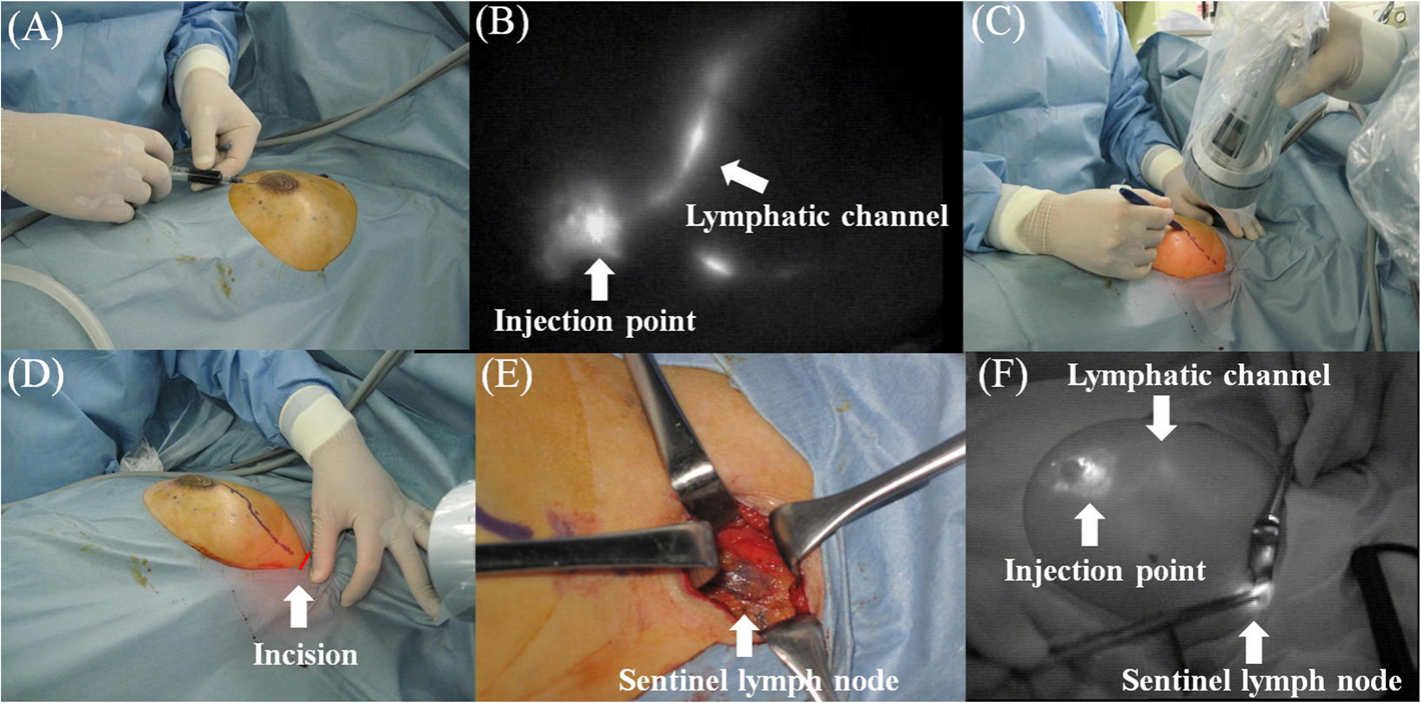
CMF method. **a** Dye injection into the sub-areolar region. **b** Fluorescence images of the lymphatic channel on the monitor. **c** Lymphatic channel marking on the skin using Photodynamic Eye images. **d** Skin incision. **e** Blue stained SLN. **f** Fluorescence image of operative field

After the induction of general anesthesia and sterilization of the operation site, a combination of 1 ml ICG (5 mg) and 1-2 ml indigo carmine (4-8 mg) was injected into the sub-areolar region (Fig. 1a). Fluorescence images were obtained using PDE, and subcutaneous lymphatic channels were detected over the skin usually within one or 2 minutes (Fig. 1b). If the lymphatic flow to the SLNs was not visible, the sub-areolar region was compressed to assess lymphatic drainage. The subcutaneous lymphatic channels were marked with a marker over the skin based on the PDE image (Fig. 1c). A 1.5-2 cm incision was made near the point of disappearance of the fluorescence in the axilla (Fig. 1d). Under shadowless surgical light conditions, the subcutaneous fat tissues were dissected, and the stained lymphatic channels were identified and carefully dissected, and traced until the first-drained lymph node was identified. Usually, one or more stained lymph nodes were identified (Fig. 1e) and resected by direct visual inspection. If the blue stained SLNs could not be identified, the operation field was directly inspected using PDE and the fluorescent lymph nodes were identified. Figure 1f illustrates a case where the blue stained lymph node could not be identified by direct visual inspection. However, using the PDE, we were able to easily identify the fluorescence of SLNs on the monitor. Fluorescence imaging was used for post-operative SLN confirmation as well. After SLN biopsy, partial resection of the mammary gland was performed through a separate incision.

### Patient characteristics

Information on patient age, body mass index (BMI) (kg/m^2^), tumor characteristics such as location, T factor, N factor, stage, presence of recurrence, and pathological type, as well as time to detection of SLNs, presence or absence of SLN identification, and number of resected SLNs were also collected. Tumor location was defined as: A,upper inner quadrant; B, lower inner quadrant; C, upper outer quadrant; D, lower outer quadrant. If the primarily involved site could not be determined due to equal extent of involvement in two or more areas, tumor location was expressed as the predominant area that occupied the resection area [25].

### Statistical analysis

The statistical analysis was done with StatMate IV (ATMS Co., Ltd., Tokyo, Japan) and Stata 14.1 (Stata Corp LP, College station, Texas, USA). We compared the baseline characteristics of the sample (age, BMI, and tumor characteristics), and intra- or post-operative findings (time to detection of SLNs, number of resected SLNs, pathological diagnosis of breast cancer, presence of estrogen and progesterone receptors, herceptine score) between patients who underwent ICG-D and CMF. The detection rates of SLNs in the two methods, including that of a subsample of SLN positive individuals based on intra-operative diagnosis, were also compared. We also examined whether the identification rate between the two methods differ by age and BMI by conducting stratified analyses. Student’s *t*-tests were used for the comparison of continuous variables. The proportion between the two groups were compared with Chi-squared tests unless it included cells ≤5, in which case Fisher’s exact tests were used. *P* < 0.05 was considered statistically significant.

### Ethical approval

Ethical approval to conduct this study was obtained from the ethics committee at the East Saitama General Hospital.

## Results

The sample consisted of 202 women. The mean (SD) age of the sample was 56.7 (12.4) years. Overall, 7.4% had ductal carcinoma in situ, and 85.6% had invasive ductal carcinoma. The ICG-D and CMF group consisted of 92 and 110 patients, respectively. The number of recurrent cases was 7 and 3 for ICG-D and CMF, respectively, but there were no cases of axillary lymph node recurrence. All pathological diagnoses of the SLNs were conducted with intra-operative frozen sections with the exception of 37 cases in the ICG-D group (permanent sections). Characteristics of patients by method of operation are illustrated in Tables 1 and 2. There were no significant differences in the baseline characteristics between the ICG-D and CMF groups (Table 1). The mean operative time to detection of SLNs was significantly shorter in the CMF group (6.65 min vs. 9.27 min; *p* < 0.001) (Table 2). The mean number of resected SLNs per patient was 2.21 (range, 0–9) for ICG-D and 1.82 (range, 0–7) for CMF (*p* = 0.061) (Table 2). The intra-operative diagnosis revealed an accessory mammary gland in two CMF cases, and these cases necessitated an additional resection of SLNs. We experienced one case of allergic shock due to ICG in the ICG-D group. However, a prompt recovery was achieved following treatment and the operation was resumed.

**Table 1:** Baseline characteristics of patients by method of operation

Characteristic		ICG-D (*n* = 92)	CMF (*n* = 110)	*P*-value
Age (years)	Mean (SD)	55.1 (12.6)	58.1 (12.1)	0.086
BMI (kg/m^2^)	Mean (SD)	22.9 (4.0)	23.2 (3.9)	0.482
Tumor location	A	26 (28.3%)	26 (23.6%)	0.731
	B	8 (8.7%)	13 (11.8%)	
	C	46 (50%)	53 (48.2%)	
	D	12 (13.0%)	18 (16.4%)	
T factor	Tis	4 (4.3%)	11 (10%)	0.352
	1	72 (78.3%)	82 (74.5%)	
	2	16 (17.4%)	17 (15.5%)	
N factor	0	76 (82.6%)	89 (80.9%)	0.756
	1	16 (17.4%)	21 (19.1%)	
Stage	0	4 (4.3%)	11 (10%)	0.264
	1	62 (67.4%)	65 (59.1%)	
	2a	26 (28.3%)	34 (30.9%)	
Recurrent cases		7 (7.6%)	3 (2.7%)	0.103

*Abbreviations*: *ICG-D* Indocyanine green dye method, *CMF* Combined mixed dye and fluorescence method, *SD* Standard deviation, *BMI* Body mass index

Data are n (%) unless otherwise stated

**Table 2:** Intra- and post-operative findings of patients by method of operation

Characteristic		ICG-D (*n* = 92)	CMF (*n* = 110)	*P*-value
Operation time (min)^a^	Mean (SD)	9.27 (4.9)	6.65 (3.9)	< 0.001
Number of resected SLNs	Mean (range)	2.21 (1–9)	1.82 (1–7)	0.061
Pathological type	Ductal carcinoma in situ	4 (4.3%)	11(10.0%)	0.112
	Invasive ductal carcinoma	83 (90.2%)	90 (81.8%)	
	Invasive lobular carcinoma	0 (0%)	4 (3.6%)	
	Special type	5 (5.4%)	5 (4.5%)	
Estrogen receptor	Positive	76 (82.6%)	97 (88.2%)	0.261
	Negative	16 (17.4%)	13 (11.8%)	
Progesterone receptor	Positive	67 (72.8%)	90 (81.8%)	0.126
	Negative	25 (27.2%)	20 (18.2%)	
Herceptine score (HER2)	Positive	15 (16.3%)	11 (10%)	0.163
	Negative	73 (79.3%)	88 (80%)	
	Unknown	4 (4.3%)	11 (10%)	

*Abbreviations*: *ICG-D* Indocyanine green dye method, *CMF* Combined mixed dye and fluorescence method, *SD* Standard deviation, *SLN* Sentinel lymph node

Data are n (%) unless otherwise stated

^a^Time to detection of sentinel lymph nodes

### Comparison between ICG-D and CMF

CMF was associated with a significantly higher SLN detection rate compared to ICG-D (96.4% vs. 83.7%; *p* = 0.003) (Table 3). There were only six CMF cases (5.5%) which required intra-operative PDE to detect SLNs. The SLN detection rate in CMF when considering these six individuals to not have had their SLNs identified was 90.9% (100/110). Among the 31 patients with confirmed SLN metastasis, ICG-D was only able to detect SLNs in 93.3% of the cases whereas CMF had a 100% detection rate (data shown only in text). For both ICG-D and CMF, axillary lymph node dissection was added when the SLN could not be identified. There were only 19 such cases (ICG-D 15 cases, CMF 4 cases). In terms of the pathological findings, age, and BMI, there were no significant difference between these 19 individuals and the rest of the sample (data shown only in text).

**Table 3:** Comparison of the identification rate of sentinel lymph nodes between ICG-D and CMF

Operation method	Identification rate	*P*-value
ICG-D	83.7% (77/92)	0.003
CMF	96.4% (106/110)	

*Abbreviations*: *ICG-D* Indocyanine green dye method, *CMF* Combined mixed dye and fluorescence method

In patients aged ≥60 years and in those with BMI ≥ 25 kg/m^2^, the SLN detection rate was particularly high in CMF as compared to ICG-D (Table 4). Specifically, in patients < 60 years of age, the SLN detection rate of CMF (vs. ICG-D) was only 1.04 times higher (94.2% vs. 90.6%; *p* = 0.716) while among the older age group, it was 1.32 times higher (98.3% vs. 74.3%; *p* < 0.001). In the case of BMI, the SLN detection rate was only 1.06 times higher (98.7% vs. 92.7%; *p* = 0.096) among those with BMI < 25 kg/m^2^ while this figure rose to 1.55 (90.3% vs. 58.3%; *p* = 0.009) among those with BMI ≥ 25 kg/m^2^.

**Table 4:** Sentinel lymph node detection rates by method of operation stratified by age and body mass index

Characteristic	Category	ICG-D	CMF	*P*-value
Age (years)	< 60	90.6% (48/53)	94.2% (49/52)	0.716
	≥60	74.3% (29/39)	98.3% (57/58)	< 0.001
BMI (kg/m^2^)	< 25	92.7% (63/68)	98.7% (78/79)	0.096
	≥25	58.3% (14/24)	90.3% (28/31)	0.009

*Abbreviations*: *ICG-D* Indocyanine green dye method, *CMF* Combined mixed dye and fluorescence method, *BMI* Body mass index

## Discussion

The conventional fluorescence method using indocyanine green is known to be an excellent SLN biopsy method with no radiation exposure, no necessity for RI facilities, and high detection and low false-negative rates [16, 19, 20]. However, this method must be performed under dim light conditions because a shadowless surgical light cannot be used as it interferes with the fluorescence of the ICG [16, 22, 23]. This is a major limitation as this implies that the image of the operative field can only be obtained via a black-and-white screen, and that only a blunt dissection of the surroundings of the fluoresecent lymph nodes can be made using a harmonic scalpel or an electrocautery. Furthermore, to manage intra-operative bleeding, the operation must be interrupted as it cannot be dealt with under dim light conditions. In addition, operation time is limited due to the rapid spread of ICG to distal lymph nodes [4, 16, 19]. Finally, in the event of an accidental damage to the lymphatic channels, the ICG leaks to the operative field and the whole operative field illuminates in the screen, rendering the identification of SLNs difficult. Thus, this method is complicated and also requires high technical skills and experience.

In order to overcome these drawbacks, we introduced the CMF method. In this method, the position of the incisional wound is determined by the fluorescence method but the surgical procedure is performed using the dye as a marker under shadowless surgical light conditions. The conventional fluorescence method using indocyanine green is used only in cases where the stained SLNs are unclear. These cases are rare and in our study, there were only 6 cases (5.5%). This method relies mainly on the dye for the detection of SLNs, with the fluoresence having an auxillary role. Thus, it is of vital importance that the dye allows for accurate detection of SLNs. In Japan, most facilities use a single dye agent such as ICG, indigo carmine and patent blue [26]. However, the SLN detection rate of ICG alone has been reported to be 71 to 84% [14, 27]. In our facility, the detection rate was 83.7% which is similar to that reported by Motomura and colleagues [27]. In order to improve detection rates, we added indigo carmine, which is covered under the Japanese national health insurance system [28].

It is also worth noting that after the advent of the fluorescence method, methods to add other dye agents to the fluorescent ICG have been reported [10, 11, 29–31]. However, these methods aim to increase the SLN detection rate by combining quite different methods [e.g., combination of RI with the dye method using two or more dye agents]. Therefore, not much importance is placed on the dye method, while some methods inject ICG and the other dye agent separately [11, 29, 30].

In our study, the SLN detection rate was 83.7% for ICG-D and 96.4% for CMF (*p* = 0.003). The identification rate of the combined method of RI and dye method has been reported to be 94–99.2% [21, 26, 32, 33]. Although false-negative rates could not be assessed in our study, the SLN identification rate of the CMF observed in our study is comparable to these figures. We believe that the higher detection rate of CMF as compared to ICG-D may be explained by the following: (1) The SLNs were more heavily stained in CMF owing to the application of mixed dye agents; (2) Confirmation of the fluorescent lymphatic channels using PDE allowed for a precise pre-operative identification of the locations of SLNs; (3) Since a skin incision could be placed right above the SLNs with CMF, the SLNs were reached from a shorter distance than ICG-D, leading to easier maneuver and shortening of operative time to detection of the SLNs. Our data show that CMF can identify more than 90% of the dye-stained SLNs, with only about 5% requiring confirmation with PDE. In contrast to the conventional fluorescence method using indocyanine green where the entire procedure including SLN biopsy must be conducted under dim light conditions, we believe that CMF is a more practical and efficient method given that SLN biopsy can be conducted under shadowless surgical light conditions in the vast majority of cases. Furthermore, it has been reported that SLNs containing only the dye or only the radioisotope exist at a probability of about 10% [34, 35]. Thus, such SLNs may potentially be overlooked by either method alone, and in theory, the identification rate is likely to increase by combining different methods. The fact that only 5.5% of CMF cases required intraoperative PDE to detect SLN reinforces the notion that a combined dye approach is likely to increase the SLN detection rate.

It has been reported that the number of dissected SLNs tends to be greater in the fluorescence method as compared to the RI method and the dye method [9, 11]. Previous studies have suggested that this may be related to the high sensitivity of the fluorescence method and the rapid spread of ICG [4, 8, 9, 16, 19]. However, in our study, there were no significant differences in the number of resected SLNs between the two methods. It may be hypothesized that this was because the CMF method mainly relies on the dye method for the detection of SLNs, and the fluorescence method only assists in the identification of the location for skin incision.

The presence or absence of post-operative axillary lymph node recurrence can be considered as an indicator to judge whether SLN biopsy was performed properly. Currently, in our study, after an average post-operative follow-up of 3.5 years (range, 0.5–7 years), there have been no cases of axillar lymph node recurrence in the 70 CMF cases which had no SLN metastasis and did not undergo axillary lymph node dissection. Thus, this leads us to believe that the accuracy of SLN biopsy in CMF is unlikely to be inferior to the RI method or other combined methods. However, the follow-up period is still short and long-term results are yet to be obtained. Since we did not perform backup axilla dissection, the false negative rate, especially for clinically occult microscopic disease, could not be definitely established. Thus, further studies of longer follow-up periods on the usefulness of the CMF method are warranted.

We also found that CMF performs particularly well as compared to ICG-D in older individuals and those with high BMI. Regarding age, several hypotheses have been proposed as for why the identification of SLNs in older women may be more difficult to achieve. For example, Cox and colleagues hypothesized that the anatomical changes related with age such as increased fatty tissue in the breast may lead to a decreased lymphatic flow [36]. Alternatively, Koizumi and colleages suggested that impaired functioning of the lymphatic channels related with lower levels of estrogen in post-menopausal and older woman may be implicated [37]. As for high BMI, several studies have reported lower SLN identification rates in obese women with the dye method and the RI method [36, 38]. Although the precise reasons are largely unknown, the high deposition of fatty tissue in patients with high BMI may induce mechanical pressure on the lymphatic vessels [37], while fatty tissue around the lymph nodes may also cause decreased flow to the lymphatic basin [36]. However, even if the lymph flow itself decreases or there is stenosis in the lymphatic channels due to obesity or age-related changes, at least a minimal flow is likely to remain. If the lymph flow is preserved, it is expected that lower molecular weight of the dye is related with easier passage through the lymphatic channel. The molecular weight of indigo carmine is 464.4, which is less than 775 in ICG [28]. Thus, molecular weight may explain why the identification rate of SLNs in the dye method increases by mixing both dyes. Even if the stained SLNs cannot be confirmed by the naked eye, there are cases where the concurrent use of the floresence method allows for SLN identification. We believe that the synergistic effect between the lower molecular weight of indigo carmine and the high sensitivity of ICG fluorescence results in an increased identification rate in CMF.

In terms of comparable methods to the CMF, recently, the HyperEye Medical System (HEMS; Mizuho Co., LTD., Tokyo, Japan) was developed. In this method, confirmation of the fluorescence, as well as SLN biopsy can be conducted through a monitor under shadowless surgical light conditions, and its utility as an alternative to the conventional fluorescence method using indocyanine green has been reported [39]. However, the procedure cannot be done under direct inspection, and the complexity of the procedure, which requires SLN biopsy under monitor guidance, has not changed. Thus, we believe that our CMF method is slightly more advanced than this method. PDE at present cannot detect lymph nodes emitting fluorescence from the body surface. However, if the fluorescent SLNs become identifiable from the body surface by improving the excitation light and filter in the future, the surgical procedure will be further simplified and the CMF method is expected to be a standard method replacing the RI method.

In terms of the limitation of the study, given that there is no overlap in the period in which ICG-D and CMF were performed, with the CMF being introduced after ICG-D, and the fact that all operations were performed by a single surgeon (NT) with a few exceptions, it is possible that the higher SLN detection rate in CMF is partially explained by increased operator experience. However, this may not be a major limitation as CMF mainly relies on the dye method for SLN detection, and unstained SLNs cannot be identified regardless of the surgical skill. We believe that this factor mainly contributed to the shortening of the operation time rather than higher SLN detection rates.

Despite the multitude of benefits associated with CMF, it also has its drawbacks which should be addressed in the future. For example, in cases where the lymph flow to the axilla cannot be confirmed by fluorescence, it is also very difficult to identify SLNs using intra-operative PDE. We were only able to identify SLNs in 4 out of 7 of these cases (57.1%) and this is an area which warrants further improvement.

## Conclusion

CMF achieves a high SLN identification rate and can reliably and easily identify SLNs under surgical light conditions. This method is safe and efficient, while it maintains a high SLN detection rate even in older patients and individuals with high BMI.

## Abbreviations

BCT: Breast Conservative Therapy
BMI: Body mass index
CMF: Combined mixed dye and fluorescence method
FI: Fluorescence imaging
HER2: Herceptine score
ICG: Indocyanine green
ICG-D: Indocyanine green dye method
PDE: Photodynamic Eye
SLNs: Sentinel lymph nodes
